# Inductive plethysmography potential as a surrogate for ventilatory measurements during rest and moderate physical exercise

**DOI:** 10.1590/bjpt-rbf.2014.0147

**Published:** 2016-03-15

**Authors:** Ramona Cabiddu, Camila B. F. Pantoni, Renata G. Mendes, Renata Trimer, Aparecida M. Catai, Audrey Borghi-Silva

**Affiliations:** 1 Laboratório de Fisioterapia Cardiopulmonar, Departamento de Fisioterapia, Universidade Federal de São Carlos (UFSCar), São Carlos, SP, Brazil; 2 Laboratório de Fisioterapia Cardiovascular, Departamento de Fisioterapia, Universidade Federal de São Carlos (UFSCar), São Carlos, SP, Brazil

**Keywords:** respiratory inductive plethysmography, movement, respiratory rate, minute ventilation, standing rest position, constant intensity exercise

## Abstract

**Background:**

Portable respiratory inductive plethysmography (RIP) systems have been validated for ventilatory assessment during resting conditions and during incremental treadmill exercise. However, in clinical settings and during field-based exercise, intensity is usually constant and submaximal. A demonstration of the ability of RIP to detect respiratory measurements accurately during constant intensity conditions would promote and validate the routine use of portable RIP devices as an alternative to ergospirometry (ES), the current gold standard technique for ventilatory measures.

**Objective:**

To investigate the agreement between respiratory variables recorded by a portable RIP device and by ES during rest and constant intensity exercise.

**Method:**

Tidal volume (V_T_), respiratory rate (RR) and minute ventilation (V_E_) were concurrently acquired by portable RIP and ES in seven healthy male volunteers during standing rest position and constant intensity treadmill exercise.

**Results:**

Significant agreement was found between RIP and ES acquisitions during the standing rest position and constant intensity treadmill exercise for RR and during the standing rest position for V_E_.

**Conclusion:**

Our results suggest that portable RIP devices might represent a suitable alternative to ES during rest and during constant submaximal exercise.

## BULLET POINTS

- We compared respiratory inductive plethysmography (RIP) and ergospirometry (ES).
- RIP advantages over ES include portability and no need for facial apparatuses.
- Agreement was found between RIP and ES for respiratory rate and ventilation.
- RIP might represent an alternative to ES during rest and submaximal exercise.

## Introduction

Reliable measurement of ventilatory parameters is essential to support research in respiratory physiology and medicine^1^. The gold standard technique is ergospirometry (ES), which provides continuous and breath-by-breath ventilatory measures. ES systems, however, involve the use of mouthpieces, which increase dead space^2^ and may be uncomfortable for the subjects^3^. In addition, they are expensive and require highly trained staff.

Respiratory inductive plethysmography (RIP) detects changes in the cross-sectional area of the rib cage and abdomen using inductive belts^4^. Respiratory volumes and timing can subsequently be obtained. Portable RIP systems incorporate belts in an elasticized vest and allow measurements to be easily obtained without a mouthpiece^5^. Evidence shows that RIP can be used to investigate respiratory mechanics in normal and symptomatic subjects^3^^,^ 6 ^,^ 7 during rest and incremental exercise^5^^,^ 6 ^,^ 8. However, during rehabilitation or field based exercise, intensity is usually submaximal and constant over a given time period^6^. A demonstration of the ability of RIP to detect respiratory measurements accurately during constant intensity conditions would promote and validate the routine use of portable RIP devices, which have the advantage of being non-invasive and easy to move in clinical settings. We aim to investigate whether agreement exists between respiratory variables, including tidal volume (V_T_), respiratory rate (RR) and minute ventilation (V_E_), recorded by a portable RIP device and by ES in healthy male subjects in the resting standing position and during constant-intensity treadmill exercise.

## Method

### Subjects

Seven apparently healthy male subjects were included. Individuals were considered healthy based on an anamnesis that included a questionnaire to record demographic data, work and health status, previous surgeries, and physical activity level. All subjects were sedentary. None of them were smokers nor used medications that might affect the measurements. A visual examination was conducted to identify thoracoabdominal alterations that might alter respiratory dynamics. Anthropometric data, including weight and height, were collected. This study was approved by the Human Research Ethics Committee of Universidade Federal de São Carlos (UFSCar), São Carlos, SP, Brazil (Process no. 145/2006), and the subjects signed an informed consent form.

### Protocol

Tests were performed in a laboratory with controlled temperature and humidity, always between 8 a.m. and 12 p.m. The day before and the day of the test volunteers were instructed to avoid stimulating drinks, refrain from physical exercise, and have an adequate night's sleep.

V_T_ [mL], RR [bpm], and V_E_ [L/min] were recorded simultaneously using a wearable RIP system (LifeShirt^Ò^, Vivometrics Inc., Ventura, CA, USA.) and an ergospirometer (CPX-D/BreezeSuite 6.4.1, Medical Graphics, St Paul, MN, USA). For the RIP system calibration, participants were asked to breathe seven times into an 800 mL plastic bag attached to a mouthpiece, filling and emptying it completely with each breath^9^. For the ES system, the carbon dioxide (CO_2_) and oxygen (O_2_) analyzers were calibrated before and after each test using a two-point measure: a calibration gas (5% CO_2_, 12% O_2_, and balance nitrogen) and a reference gas (room air after ATPS [ambient temperature and pressure saturated] to STPD [standard temperature and pressure, dry] correction).

Heart rate (HR) was measured while standing before and after exercise. Ventilatory variables were collected using ES and RIP during 5 minutes of standing rest. Afterwards, subjects started the treadmill exercise. Speed was increased at 30-second intervals until the HR was 20 beats faster than the resting value. This speed was maintained and ventilatory variables were collected using ES and RIP during 6 minutes.

### Data analysis

An *a posteriori* power analysis (G*Power, F. Faul, Universität Kiel, Germany) was performed. Considering a p=0.05, the statistical power of this study is 60%.

Data analysis (MATLAB^Ò^, The Mathworks Inc., Natick, MA, USA) was performed on 4.5 minutes of signal acquired during rest (excluding the first 30 seconds) and on 5.5 minutes of signal acquired during exercise (excluding the first 30 seconds). Time accordance between RIP and ES breath-by-breath values was verified.

For each parameter and for each condition, the seven signals coming from all volunteers were averaged. Bland-Altman plot and Spearman correlation analyses were performed between RIP and ES values. Normality of distribution was verified using the Kolmogorov-Smirnov test. Significant correlations (p-value ≤0.05) were considered weak when 0≤r<0.3, moderate when 0.3≤r<0.7, and strong when r≥0.7.

## Results

Mean age was 25 years (SD=3), mean weight was 73 Kg (SD=12), and mean height was 177 cm (SD=6). Average ventilatory parameters obtained during rest and exercise using ES and RIP are presented in Table 1.

**Table 1.** Average values of ventilatory parameters obtained during resting standing position and treadmill exercise using ES and RIP.

	**Resting standing position**	**Treadmill exercise**		
	**ES**	**RIP**	**ES**	**RIP**
V_T_ [mL]	487.0±34.9	471.0±131.4	1030.5±134.5	1017.2±173.1
RR [bpm]	16.2±3.2	16.4±3.7	23.3±3.8	24.8±2.6
V_E_ [L/min]	7.8±1.4	9.2±5.2	23.7±5.3	24.3±2.8

Data are presented as mean±SD. V_T_: tidal volume; RR: respiratory rate; V_E_: minute ventilation.

**Figure 1.**
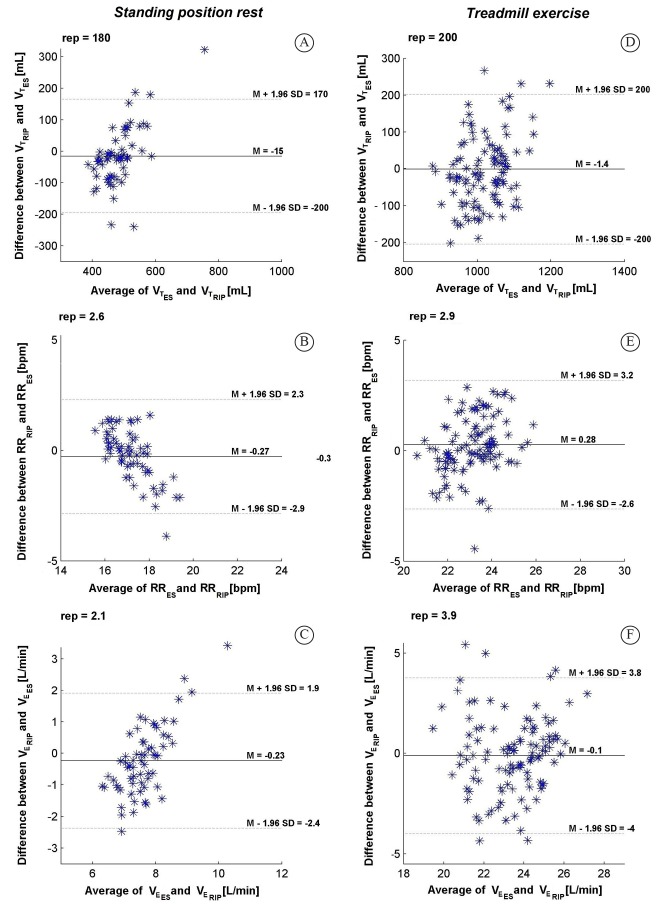
Bland-Altman plots showing agreement of the mean differences between (A) V_T_, (B) RR, and (C) V_E_ average signals calculated over the signals measured using ES and RIP during the resting standing position and between (D) V_T_, (E) RR, and (F) V_E_ average signals calculated over the signals measured using ES and RIP during the treadmill exercise. Rep: reproducibility index; M: mean; SD: standard deviation.

**Figure 2.**
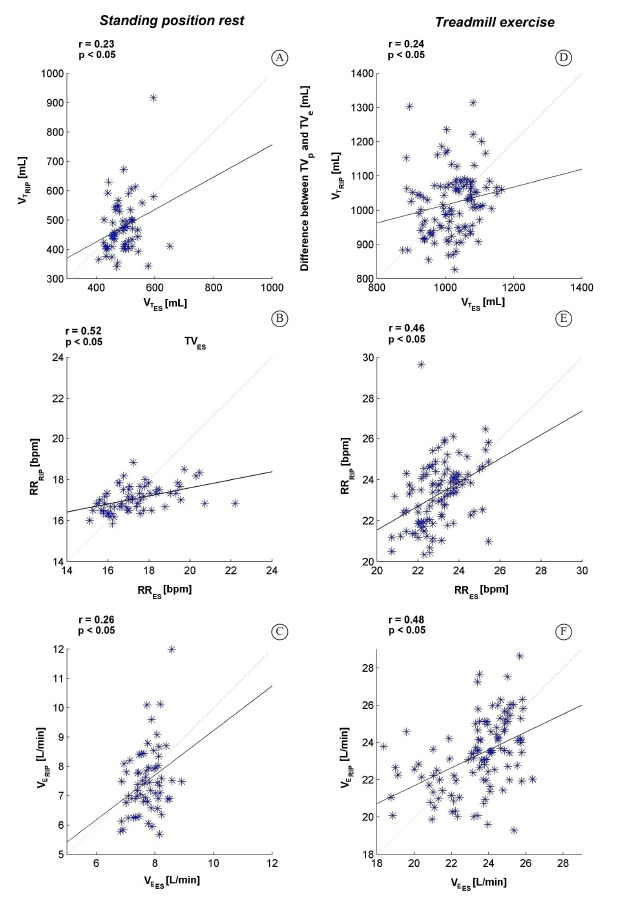
Correlation plots between (A) V_T_, (B) RR, and (C) V_E_ average signals calculated over the signals measured using ES and RIP during the standing rest position and between (D) V_T_, (E) RR and (F) V_E_ average signals calculated over the signals measured using ES and RIP during the treadmill exercise. r, correlation coefficient. For each correlation, regression lines are presented.

Average Bland-Altman and correlation results obtained for the whole group, during rest and exercise, are shown in Figure 1 and in Figure 2, respectively.

## Discussion

The present study aimed to evaluate the potential of portable RIP as an alternative to ES during rest and steady-state treadmill exercise. Significant correlations were found between RIP and ES acquisitions; good agreement was found for RR, during rest and exercise, and V_E_, during rest.

V_T_ values recorded using RIP and ES presented low agreement (rest: bias=15 mL; rep=180 mL; exercise: bias=1.4 mL; rep=200 mL); however, significant but weak correlations were found (rest: r=0.23; exercise: r=0.25). This is in line with Hollier et al.^1^, who performed RIP and ES to measure ventilatory variables in sitting position and during two breathing tests on untreated obesity hypoventilation syndrome patients and controls. Our results suggest that RIP translates into a qualitative measurement related to ES, despite a reduced agreement between the methods. RIP-ES agreement was acceptable for RR both during rest (r=0.52; bias=0.27 bmp; rep=2.6 bpm) and exercise (r=0.46; bias=0.28 bmp; rep=2.9 bpm). Correlation between RIP and ES for V_E_ values was significant during rest (r=0.23) and exercise (r=0.45). Agreement was acceptable during rest (bias=0.23 L/min; rep=2.1 L/min) and low during exercise (bias=1 L/min; rep=3.9 L/min).

In summary, our results confirm that significant, quantitative agreement exists between RIP and ES acquisitions for RR, during rest and constant intensity treadmill exercise, and for V_E_, during rest. To choose between one or the other, health professionals should consider what measurements are needed and that, due to the absence of airway instrumentation, RIP will probably be better tolerated^3^.

Limitations of this study include a low number of subjects, which might help explain why partial disagreement was observed with Clarenbach et al.^5^, who showed that RIP-ES agreement was similar for all indices in healthy volunteers and cardiorespiratory patients during progressive treadmill exercise to exhaustion. Implementation of a different kind of exercise (constant, submaximal intensity conditions, which increase the respiratory pattern variability^10^) might also have influenced results. It is also worth noticing that differences in the system calibration might have influenced measurements as well. Moreover, our investigation is limited to the study of healthy, young men during standing position and constant treadmill exercise and may not apply to other populations, as elderly people or women. In conclusion, our results suggest that RIP and ES can be used interchangeably in healthy, young male subjects to evaluate RR quantitatively during rest and constant intensity treadmill exercise and V_E_ during resting conditions.

## References

[1] Hollier CA, Harmer AR, Maxwell LJ, Menadue C, Willson GN, Black DA (2014). Validation of respiratory inductive plethysmography (LifeShirt) in
obesity hypoventilation syndrome. Respir Physiol Neurobiol.

[2] Perez W, Tobin MJ (1985). Separation of factors responsible for change in breathing pattern
induced by instrumentation. J Appl Physiol.

[3] Bloch KE, Li Y, Sackner MA, Russi EW (1997). Breathing pattern during sleep disruptive snoring. Eur Respir J.

[4] Cohn MA, Rao AS, Broudy M, Birch S, Watson H, Atkins N (1982). The respiratory inductive plethysmograph: a new non-invasive monitor
of respiration. Bull Eur Physiopathol Respir.

[5] Clarenbach CF, Senn O, Brack T, Kohler M, Bloch KE (2005). Monitoring of ventilation during exercise by a portable respiratory
inductive plethysmograph. Chest.

[6] Witt JD, Fisher JR, Guenette JA, Cheong KA, Wilson BJ, Sheel AW (2006). Measurement of exercise ventilation by a portable respiratory
inductive plethysmograph. Respir Physiol Neurobiol.

[7] Grossman P, Wilhelm FH, Brutsche M (2010). Accuracy of ventilatory measurement employing ambulatory inductive
plethysmography during tasks of everyday life. Biol Psychol.

[8] Eberhard A, Calabrese P, Baconnier P, Benchetrit G (2001). Comparison between the respiratory inductance plethysmography signal
derivative and the airflow signal. Adv Exp Med Biol.

[9] Cancelliero-Gaiad KM, Ike D, Pantoni CB, Borghi-Silva A, Costa D (2014). Respiratory pattern of diaphragmatic breathing and pilates breathing
in COPD subjects. Braz J Phys Ther.

[10] Pfaltz MC, Grossman P, Michael T, Margraf J, Wilhelm FH (2010). Physical activity and respiratory behavior in daily life of patients
with panic disorder and healthy controls. Int J Psychophysiol.

